# Increased volume of the left hippocampal dentate gyrus after 4 weeks of bright light exposure in patients with mood disorders: a randomized controlled study

**DOI:** 10.1038/s41398-023-02688-9

**Published:** 2023-12-15

**Authors:** Hirofumi Hirakawa, Takeshi Terao, Koji Hatano, Masanao Shirahama, Tsuyoshi Kugimiya, Kentaro Kohno, Hiroyuki Matsuta, Tsuyoshi Shimomura, Minoru Fujiki

**Affiliations:** 1 Department of Neuropsychiatry, Oita University Faculty of Medicine, Oita, Japan; 2 Department of Neurosurgery, Oita University Faculty of Medicine, Oita, Japan; 3 Hospital informatics center, Oita University Faculty of Medicine, Oita, Japan

**Keywords:** Bipolar disorder, Depression

## Abstract

Bright light exposure (BL) induces neurogenesis in the rat hippocampal dentate gyrus (DG). We had previously conducted a randomized controlled trial (RCT) in which a 4-week period of BL in healthy participants resulted in increased volume of the left DG-head. This study aimed to investigate the effects of BL on the DG in patients with mood disorders. A 4-week RCT was conducted in which patients with mood disorders were randomly assigned to either a BL group (10,000 lx) or dim light exposure group (DL group; 50 lx). All patients underwent clinical assessment and magnetic resonance imaging at baseline and after the intervention. The study registration number is UMIN000019220. Our final sample included 24 patients (BL group, *n* = 12; DL group, *n* = 12). A significant effect of time and group was detected in the volumes of the left DG-head (F (1, 22) = 11.6, partial η2 = 0.35, *p* = 0.003) and left DG-total (left DG-total = left DG-head + left DG-body; [F (1, 22) = 6.5, partial η2 = 0.23, *p* = 0.02]). Additionally, the BL group demonstrated a significant increase in the volume of the left DG-head (95% CI: −5.4 to −1.6, d = 1.2, *p* = 0.002) and left DG-total (95% CI: −6.3 to −1.5, d = 1.06, *p* = 0.005) as well as a positive correlation between the percentage change in the volume of the left DG-total and the percentage change in the scores of the mood visual analog scale (r = 0.58, *p* = 0.04). In conclusion, our study results suggest that compared to DL, BL leads to a significantly greater increase in the left DG volume in patients with mood disorders. This increase in the left DG volume may be associated with mood improvement in the patients.

## Introduction

Bright light therapy (BLT) is an established standard treatment for seasonal affective disorder and has been explored for its efficacy in treating other conditions such as major depressive disorder (MDD) and bipolar depression [1]. BLT is a non-pharmacological intervention that is safe, well-tolerated, and low-cost, with a favorable risk-to-benefit ratio [2]. Several hypotheses about BLT mechanisms have been proposed, including the modulation of circadian rhythms by the suprachiasmatic nucleus, regulation of melatonin secretion, advancement of circadian rhythm, and interactions with serotonin [1]. However, the precise mechanism of BLT remains to be elucidated. The hippocampus is a fundamental brain region involved in neurogenesis, whereby functional neurons are continuously generated from neural stem cells throughout life [3, 4]. In particular, neurogenesis occurs in the adult hippocampal dentate gyrus (DG) [4]. Prior research has revealed that stress inhibits adult hippocampal neurogenesis in both animals and humans, providing a cellular basis for understanding depression-related impairment of neural plasticity [5]. Antidepressant therapy has been shown to restore neurogenesis in the hippocampus and normalize behavior in animal models of depression, suggesting that deficits in hippocampal neurogenesis could serve as a promising target for depression therapy [5]. A study using 5-bromo-2′-deoxyuridine as a marker of cell proliferation reported that 4 weeks of BLT induced neurogenesis in the adult rat hippocampal DG [6], thus prompting our hypothesis that BLT may also induce neurogenesis in the human hippocampus, particularly in the DG. To explore our hypothesis, we recently conducted a randomized controlled trial which revealed that a 4-week period of BLT in healthy participants resulted in an increase in the volume of the left hippocampal DG-head [7]. Our study was the first investigation into the relationship between BLT and volumetric changes in the human hippocampal DG, and the results suggested that neurogenesis may be induced by BLT in the human DG [7]. Considering that the participants in our previous study were healthy individuals, the aim of the current randomized controlled trial was to further investigate the effects of light therapy (BLT vs. dim light therapy) on the hippocampal DG and the correlation between alterations in DG volume and the amelioration of depressive symptoms in a sample population of patients with mood disorders.

## Subjects and methods

### Study design

**Fig. 1:**
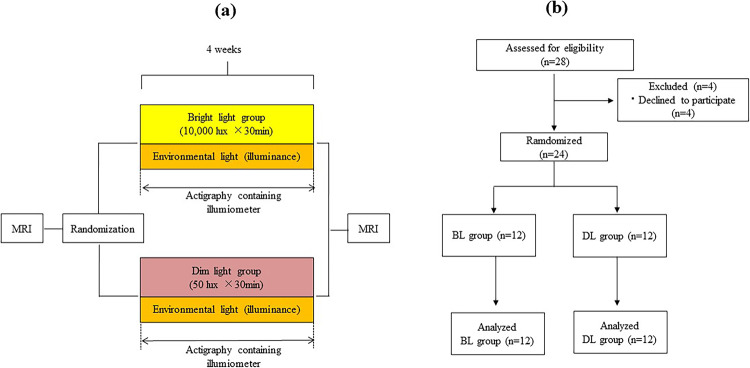
Trial protocol and profile. **a** Trial protocol. Patients were randomly allocated to either bright light exposure group (BL group) or dim light exposure group (DL group), utilizing the envelope method in a 1:1 ratio. Both BL group and DL group had an actigraphy system to measure illuminance. BL group received bright light (10,000 lux) derived from the light therapy device and DL group received dim light derived from the light therapy device fixed with 50 lux. Light exposure had done for 30 min between 7:00 a.m. and 7:30 a.m. every morning for 4 weeks. **b** Trial profile. Twenty-eight patients were assessed for eligibility, and four declined to participate in the study. The remaining 24 patients were diagnosed with mood disorder and randomized to either the BL or DL group, with 12 patients in each group. All patients completed the protocol, resulting in a final sample size of 24.

A randomized controlled trial spanning a duration of 4 weeks was conducted among patients diagnosed with mood disorders who were randomly assigned to either a bright light exposure group (BL group) or a dim light exposure group (DL group). Eligible participants were recruited from the patients visiting Oita University Hospital. Written informed consent was obtained from all the patients. Patient participation was withdrawn if they revoked their consent, a significant adverse event occurred, or they failed to adhere to the study schedule. We confirm that the methodology utilized in this study was in accordance with the ethical standards and relevant national and institutional guidelines for human experimentation as well as the principles outlined in the 1975 Helsinki Declaration (revised in 2008). The study protocol was approved by the Institutional Review Board of Oita University Faculty of Medicine (B15-019). The study registration number is UMIN000019220. The study was initiated on 16 October 2015 and completed on 19 February 2021. Figure 1a illustrates the trial protocol.

### Participants

The inclusion criteria for the participants included a diagnosis of MDD or bipolar disorder (BP) as per the Diagnostic and Statistical Manual of Mental Disorders (DSM)-IV-TR or DSM-5 criteria, age 20 years or older, right-handedness, and psychotropic medication unchanged for at least 1 month prior to enrollment. All patients were screened using the Mini-International Neuropsychiatric Interview (MINI) to identify coexisting psychiatric disorders. The exclusion criteria included any serious physical ailments, cognitive impairment affecting decision-making abilities, inability to provide consent, pregnancy, or lactation.

### Randomization, masking, and intervention

The patients were randomly allocated to either the BL or DL group using the envelope method in a 1:1 ratio. The random allocation sequence was generated by HH and MS, and HH enrolled the patients. The participants then drew envelopes and were assigned to the BL or DL group. The outcome of the randomization was not concealed. The BL and DL groups were exposed to light therapy devices emitting 10,000 lx (Bright Light Me +; Solartone, Japan) and 50 lx (Wake-Up Light HF3520; Royal Philips, Netherlands), respectively. The daily light exposure protocol was administered for 30 min between 7:00 and 7:30 a.m. every morning for 4 weeks. At the inception of the study, participants were instructed to maintain their lifestyle unchanged throughout the study’s duration. Also, the patients were continued on the same medications and dosages throughout the study period.

### Procedures, image acquisition, and processing

All patients underwent clinical assessments using the 17-item Hamilton Depression Rating Scale (HAM-D), Beck Depression Inventory (BDI), Young Mania Rating Scale (YMRS), and a visual analog scale (VAS) of mood (using a 100 mm linear scale, where 0 mm indicates the worst feeling and 100 mm represents the best feeling) in the morning at baseline (pre) and after a 4-week period of light exposure (post). An actigraphy system (Actiwatch 2; Respironics Inc., USA) was employed to measure environmental light, including both indoor and outdoor light, during the study period for both groups. Magnetic resonance imaging (MRI) data were collected using a 3 T MRI scanner (MAGNETOM Verio, Siemens or MAGNETOM Skyra Fit, Siemens), with a gradient strength of 45 mT/m and 32-channel array head coil. T1-weighted structural images were acquired using a 3-D magnetisation-prepared rapid gradient-echo sequence in the sagittal plane. Images were automatically processed with the longitudinal stream in FreeSurfer 7.1.1 to extract reliable volume estimate [8]. Longitudinal hippocampal subfield segmentation was generated via the FreeSurfer 7.1.1 hippocampal subfields module to evaluate the volume of the bilateral granule cell and molecular layers of the DG-head and -body [9, 10].

### Sample size

This was the first randomized controlled trial to investigate the effects of light therapy on the DG in patients with mood disorders. Therefore, statistical considerations could not be used to estimate the sample size.

### Statistical analysis

The demographic characteristics of all participants were analyzed using exploratory data analysis. The demographic characteristics of the BL and DL groups were compared using two-sample *t* tests and chi-square tests. The primary outcome of interest was the volumetric changes in the hippocampal DG. The volume of the DG-total was computed by summing the volumes of the DG-head and -body, and we used the volumes of the DG-total, DG-head, and DG-body for analysis. To assess the influence of age and sex on the baseline volume of the DG, we employed Pearson’s correlation coefficient and conducted two-sample *t* tests. The effects of different light exposures (i.e. BL group vs. DL group) on DG volumes were examined using repeated-measures analysis of variance (ANOVA) in a split-plot factorial design. As this study investigated the effect of light on the DG, we adjusted for age, sex, and log-transformed environmental light as covariates. Furthermore, due to the enrollment of patients with mood disorders, this study incorporated two mixed diagnosis cases of MDD and BP and included their diagnoses as covariates in the analysis. Post-hoc analysis via paired t-tests was used to further examine the volumetric changes in the hippocampal DG in each group. We did not perform the Bonferroni correction for family wise errors because the sample size in this study was relatively small. The significance level was set at *p* < 0.05. Furthermore, participants were prescribed psychotropic medications for the duration of the study. Considering that lithium is generally accepted for its neurotrophic and neuroprotective properties and its ability to increase hippocampal volume, we performed a subgroup analysis excluding those receiving lithium. The secondary outcome was the change in clinical symptoms as measured by the questionnaires, which were analyzed using repeated-measures ANOVA in a split-plot factorial design and via paired t-tests for the post-hoc analysis. Especially, to confirm the light effect on depression, two important items (depressed mood, work and interests) in the 17-item HAM-D were analyzed. The significance level was set at *p* < 0.05. Additionally, the correlation between alterations in the hippocampal DG volume and alterations in the depression symptoms evaluated using HAM-D, BDI, and VAS of mood was assessed. The percentage change (denoted as %Δ) was determined as the rate of alteration. For example, the %Δ in the left DG-head volume was computed as ([post volume of the left DG-head—pre volume of the left DG-head]/pre volume of the left DG-head) ×100. Pearson’s correlation coefficient was used to analyse the outcomes. The significance level was set at *p* < 0.05. Statistical analyses of the demographic data were conducted using IBM SPSS Statistics version 27.0 (IBM SPSS Inc., Chicago, IL, USA).

## Results

### Participants and baseline characteristics

Twenty-eight patients were assessed for eligibility, out of which four declined to participate in the study. The remaining 24 participants were diagnosed with mood disorders and randomized to either the BL or DL group, leading to 12 patients in each group. All enrolled patients completed the protocol, and the final sample size consisted of 24 participants (Fig. 1b, Supplementary Table 1). The average age of the participants in the BL group was 37.7 ± 12.1 years, among which six participants were males. The BL group included seven participants who were diagnosed with MDD and five with BP. In case of the DL group, the average age of the participants was 38.3 ± 11.4 years, with six male participants. In this group, 10 participants were diagnosed with MDD and two with BP. Baseline assessment revealed that patients in both groups had mild depression, as indicated by their HAM-D and BDI scores, but they were not in a manic or hypomanic state, as demonstrated by their YMRS scores. Furthermore, the baseline volumes of the hippocampal DG-head, -body, and -total did not differ significantly between the two groups. The demographic characteristics, psychiatric comorbidities, and psychotropic medications were similar between the BL and DL groups (Table 1). Also, there were no significant correlations or differences between the baseline volume of the DG and either age or sex (Supplementary Table 2, Supplementary Table 3).

**Table 1:** The demographic characteristics of patients with mood disorder.

	Patients with mood disorder (*n* = 24)		*p* value
	BL group (*n* = 12)	DL group (*n* = 12)	
Age mean (s.d.)	37.7 (12.1)	38.3 (11.4)	0.89
Sex (Male, Female)	6, 6	6, 6	1
Diagnosis	MDD: 7, BP: 5	MDD: 10, BP: 2	0.37
Comorbidity	Panic: 2, SAD:1	Panic: 5, SAD: 2, GAD: 1	
Psychotropics	Antidepressant:10 (Esci:2, Mirt:2, Sert:1, Paro:2, Dulo:1, Venl:1, Traz:1), Mood stabilizer:6 (LTG:3, Li:3), Antipsychotics:7 (QUE:4, ARI:1, LEV:2), Hypnotics:9	Antidepressant:16 (Esci:4, Dulo:3, Mirt:6, Sert:1, Paro:1, Dosu:1), Mood stabilizer:7 (LTG:4, Li:2, VPA:1), Antipsychotics:6 (QUE:2, ARI:2, OLA:1, SUL:1), Hypnotics:10	
Season	Spring:1, Summer:4, Autumn:4, Winter:3	Spring:1, Summer:3, Autumn:3, Winter:5	0.85
Environmental light (s.d.) lux	168.3 (60.1)	129.8 (52.2)	0.11
Log-transformed environmental light (s.d.)	2.20 (0.17)	2.08 (0.20)	0.11
HAM-D (s.d.)	9.1 (3.6)	9.83 (4.0)	0.64
BDI (s.d.)	18.6 (10.9)	20.9 (10.0)	0.59
YMRS (s.d.)	1.3 (1.4)	1.0 (1.0)	0.62
VAS of mood (s.d.)	45.5 (13.8)	42.5 (15.2)	0.62
DG Volume mm^3^ mean (s.d.)			
Left DG-head	153.5 (23.0)	156.1 (17.9)	0.76
Left DG-body	131.8 (14.9)	132.0 (20.9)	0.98
Left DG-total	285.3 (33.4)	288.2 (34.2)	0.84
Right DG-head	161.0 (21.4)	158.9 (15.3)	0.77
Right DG-body	139.1 (17.7)	132.5 (17.6)	0.37
Right DG-total	300.3 (35.3)	291.4 (25.5)	0.49

*ARI* Aripiprazole, *BDI* Beck Depression Inventory, *BP* bipolar disorder, *BL group* Bright light exposure group, *DG* dentate gyrus, *DL group* Dim light exposure group, *Dosu* Dosulepin, *Dulo* Duloxetine, *Esci* Escitalopram, *GAD* Generalized anxiety disorder, *HAM-D* Hamilton Depression Rating Scale, *MDD* Major Depressive Disorder, *Mirt* Mirtazapine, *LEV* Levomepromazine, *Li* Lithium, *LTG* Lamotrigine, *OLA* Olanzapine, *Panic* panic disorder, *Paro* paroxetine, QUE Quetiapine, *Sert* Sertraline, *SUL* sulpiride, *SAD* social anxiety disorder, *s.d.* standard deviation, *Traz* Trazodone, *VPA* Valproate, *Venl* Venlafaxine, *VAS* Visual Analog Scale, *YMRS* Young Mania Rating Scale.

### Effect of light on the volume of the hippocampal DG in patients with mood disorders

**Fig. 2:**
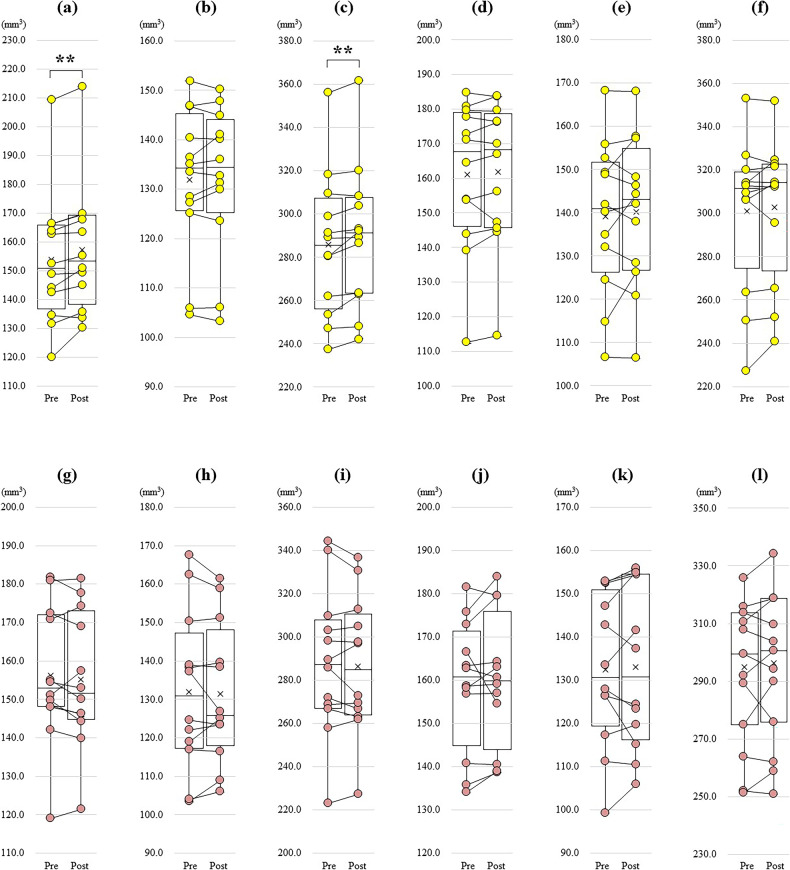
Change of the dentate gyrus (DG) volume in the bright light exposure (BL) group and dim light exposure (DL) group. **a** The left DG-head volume significantly increased (*p* = 0.002) in BL. **b** The left DG-body volume did not change significantly in BL. **c** The left DG-total volume significantly increased (*p* = 0.005) in BL. **d** The right DG-head volume did not change significantly in BL. **e** The right DG-body volume did not change significantly in BL. **f** The right DG-total volume did not change significantly in BL. **g** The left DG-head volume did not change significantly in DL. **h** The left DG-body volume did not change significantly in DL. **i** The left DG-total volume did not change significantly in DL. **j** The right DG-head volume did not change significantly in DL. **k** The right DG-body volume did not change significantly in DL. **l** The right DG-total volume did not change significantly in DL. *Indicates that the *p* values < 0.05, ** Indicates that the *p* values < 0.01.

Time and group had significant effects on the volumes of the left DG-head (F (1, 22) = 11.6, partial η2 = 0.35, *p* = 0.003) and left DG-total (F (1, 22) = 6.5, partial η2 = 0.23, *p* = 0.02). However, no such effects were found in the volumes of the other subregions of the hippocampal DG, including the left DG-body, right DG-head, right DG-body, and right DG-total (Supplementary Table 4). After adjusting for potential confounders, such as age, sex, and log-transformed environmental light, significant effects of time and group persisted in the volumes of the left DG-head and left DG-total, whereas no significant effects were observed in the volumes of the other subregions of the hippocampal DG (Supplementary Table 4). Additionally, after controlling for the influence of diagnosis (i.e. MDD or BP) as a covariate in the aforementioned analysis, significant effects of time and group persisted in the volumes of the left DG-head and left DG-total, while no such significant effects were observed in the volumes of the other subregions of the hippocampal DG (Supplementary Table 4). Post-hoc analysis to investigate the volumetric changes in the hippocampal DG was performed using paired t-tests. In the BL group, the volumes of the left DG-head (95% CI: −5.4 to −1.6, d = 1.2, *p* = 0.002) and left DG-total (95% CI: −6.3 to −1.5, d = 1.06, *p* = 0.005) were significantly increased following 4 weeks of light exposure, whereas no significant alterations were observed in the volumes of the other subregions of the hippocampal DG (Table 2, Fig. 2). In contrast, the DL group demonstrated no significant volumetric changes in any subregions of the hippocampal DG after 4 weeks of light exposure (Table 2, Fig. 2). Furthermore, we excluded three patients from the BL group and two patients from the DL group who were administered lithium and performed a subgroup analysis involving the remaining nine patients from the BL group and 10 from the DL group. The results showed similarity to those obtained prior to the exclusion of cases who administered lithium treatment (Supplementary Tables 5, 6).

**Table 2:** Questionnaire and volumetric change of hippocampal dental gyrus after bright or dim light exposure in patients with mood disorder.

	Patients with mood disorder									
	BL group pre	BL group post	95% CI	d	*p* value	DL group pre	DL group post	95% CI	d	*p* value
Questionnaire										
HAM-D (s.d.)	9.1 (3.6)	4.9 (3.5)	1.8–6.5	1.16	0.002 *	9.8 (4.0)	7.6 (3.7)	0.53–4.0	0.83	0.015 *
BDI (s.d.)	18.6 (10.9)	7.6 (8.1)	7.6–14.4	2.05	0.00002 *	20.9 (9.9)	14.3 (9.1)	3.2–10.2	1.21	0.002 *
YMRS (s.d.)	1.3 (1.4)	0.5 (0.8)	0.03–1.5	0.66	0.043 *	1.0 (1.0)	0.5 (0.8)	−0.01–1.0	0.63	0.05
VAS of mood (s.d.)	45.5 (13.8)	62.5 (15.2)	−25.2–−8.7	1.31	0.0008 *	42.5 (15.2)	50.5 (8.6)	−16.3–0.4	0.61	0.06
DG Volume mm^3^ mean (s.d.)										
Left DG-head	153.5 (23.0)	157.0 (22.7)	−5.4–−1.6	1.2	0.002 *	156.1 (17.9)	155.0 (17.7)	−1.2–3.4	0.31	0.32
Left DG-body	131.8 (14.9)	132.2 (15.0)	−1.7–1.0	0.17	0.55	132.0 (20.9)	131.6 (18.4)	−2.5–3.4	0.09	0.75
Left DG-total	285.3 (33.4)	289.2 (33.2)	−6.3–−1.5	1.06	0.005 *	288.2 (34.2)	286.7 (31.9)	−2.5–5.5	0.24	0.42
Right DG-head	161.0 (21.4)	162.1 (21.0)	−2.9–1.1	0.27	0.35	158.9 (15.3)	159.9 (15.6)	−4.4–2.3	0.2	0.51
Right DG-body	139.1 (17.7)	140.3 (17.5)	−4.7–2.4	0.2	0.49	132.5 (17.6)	133.1 (18.9)	−4.4–3.0	0.12	0.68
Right DG-total	300.3 (35.3)	302.3 (33.0)	−6.2–2.2	0.32	0.31	291.4 (25.5)	293.1 (26.5)	−6.8–3.3	0.22	0.46

*BDI* Beck Depression Inventory, *BL group* Bright light exposure group, *CI* confidence interval, *DG* Dentate gyrus, *DL group* Dim light exposure group, *HAM-D* Hamilton Depression Rating Scale, *s.d.* standard deviation, *VAS* visual Analog Scale, *YMRS* Young Mania Rating Scale.

*Indicates that the *p* values < 0.05, ** Indicates that the *p* values < 0.01.

### Effect of light on clinical symptoms

A significant tendency was observed for the effects of time and group on BDI scores, but no significant correlation was found between time and group effects and the scores on the HAM-D, YMRS, or VAS of mood. After adjusting for variables, such as age, sex, and log-transformed environmental light, no significant effects of time or group were found on the BDI, HAM-D, YMRS, or VAS of mood (Supplementary Table 4). Significant effects of time were observed in all questionnaires (i.e. HAM-D, BDI, YMRS, and VAS of mood), however, they were not significant after adjusting for variables (Supplementary Table 4). The HAM-D, BDI, and YMRS scores significantly decreased while the VAS of mood score significantly increased in the BL group after 4 weeks of intervention (Table 2). In the DL group, the HAM-D and BDI scores significantly decreased, whereas the YMRS scores demonstrated a decreasing tendency and the VAS of mood scores showed an increasing tendency after 4 weeks of intervention (Table 2). Thus, both BL and DL improved depressive symptoms in patients with mood disorders. In addition, analysis of the two items (depressed mood, work and interests) in the 17-item HAM-D revealed that there were significant effects of time and group interaction for work and interests, however, there were no significant effects for depressed mood (Supplementary Table 7). The scores of the depressed mood and work and interests were significantly decreased in the BL group, whereas not significantly decreased in the DL group (Supplementary Table 8). Therefore, compared to DL, BL tended to have a greater effect on improving subjective depressive symptoms in patients with mood disorders. No apparent adverse effects were observed in either group during the study period.

### Correlation between alterations in the volume of the hippocampal DG and alterations in the scores of the depression symptoms questionnaires

In the BL group, a significant positive correlation was observed between the %Δ in the volume of the left DG-total and the %Δ in the VAS of mood scores (r = 0.58, *p* = 0.04). However, no significant correlation was found between the %Δ in the volume of the hippocampal DG and the %Δ in the HAM-D or BDI scores (Table 3). In the DL group, a significant positive correlation was found between the %Δ in the volume of the left DG-body and the %Δ in the HAM-D scores (r = 0.64, *p* = 0.02). In contrast, no significant correlation was observed between the %Δ in the volume of the hippocampal DG and the %Δ in the BDI or VAS of mood scores (Table 3).

**Table 3:** The correlation between alterations in the volume of the dentate gyrus in the hippocampus and alterations in the depression symptoms questionnaire in the bright light exposure group and dim light group.

	%Δ HAM-D	%Δ BDI	%Δ VAS of mood
BL group			
%Δ Left DG-head	r = −0.33, *p* = 0.29	r = −0.21, *p* = 0.52	r = 0.38, *p* = 0.23
%Δ Left DG-body	r = 0.37, *p* = 0.23	r = 0.52, *p* = 0.09	r = 0.51, *p* = 0.09
%Δ Left DG-total	r = −0.06, *p* = 0.85	r = 0.12, *p* = 0.71	r = 0.58, *p* = 0.04 *
%Δ Right DG-head	r = −0.19, *p* = 0.56	r = 0.17, *p* = 0.61	r = 0.09, *p* = 0.77
%Δ Right DG-body	r = 0.04, *p* = 0.91	r = 0.12, *p* = 0.71	r = 0.5, *p* = 0.1
%Δ Right DG-total	r = −0.09, *p* = 0.78	r = 0.14, *p* = 0.66	r = 0.46, *p* = 0.13
DL group			
%Δ Left DG-head	r = −0.12, *p* = 0.70	r = 0.01, *p* = 0.98	r = 0.05, *p* = 0.88
%Δ Left DG-body	r = 0.64, *p* = 0.02 *	r = 0.46, *p* = 0.13	r = −0.48, *p* = 0.11
%Δ Left DG-total	r = 0.4, *p* = 0.20	r = 0.33, *p* = 0.29	r = −0.34, *p* = 0.29
%Δ Right DG-head	r = −0.24, *p* = 0.46	r = −0.45, *p* = 0.15	r = −0.12, *p* = 0.70
%Δ Right DG-body	r = 0.15, *p* = 0.64	r = 0.16, *p* = 0.61	r = 0.002, *p* = 0.99
%Δ Right DG-total	r = −0.03, *p* = 0.92	r = −0.15, *p* = 0.63	r = −0.07, *p* = 0.83

*BDI* Beck Depression Inventory, *BL group* Bright light exposure group, *DG* Dentate gyrus, *DL group* Dim light exposure group, *HAM-D* Hamilton Depression Rating Scale, *s.d.* standard deviation, *VAS* visual Analog Scale, *YMRS* Young Mania Rating Scale.

*Indicates that the *p* values < 0.05.

## Discussion

Our randomized study demonstrated that time and group had significant effects on the volumes of the left DG-head and left DG-total. These effects remained even after adjusting for confounding factors, such as age, sex, log-transformed environmental light, and diagnoses. Furthermore, a significant increase in the volumes of the left DG-head and left DG-total in the BL group, whereas no significant changes were detected in the volume of the DG in the DL group. Moreover, the %Δ in the volume of the left DG-total was positively associated with the %Δ in the VAS of mood scores. To the best of our knowledge, this is the first study to investigate the relationship between BLT and longitudinal volumetric changes in the hippocampal DG as well as examine the correlation between alterations in DG volume and amelioration of depressive symptoms in patients with mood disorders.

The DG of the hippocampus is a key brain region involved in stress response, depression pathology, and antidepressant response, making it a useful target for interventional therapy for mood disorders, particularly via the modulation of adult hippocampal neurogenesis [11]. Mood stabilizers such as lithium, antidepressants (e.g. selective serotonin reuptake inhibitors [SSRIs]), and electroconvulsive therapy, increase adult hippocampal neurogenesis [11–16]. In our study, significant volumetric changes were observed in the hippocampal left DG-head and left DG-total in the BL group but not in the DL group. These findings suggest that BL induces neurogenesis in the left DG. Furthermore, the increase in DG volume was found to be specifically localized in the DG-head rather than in the DG-body. Pathological investigations of postmortem samples of adult humans with MDD have demonstrated that antidepressants, including SSRIs and tricyclic antidepressants, increase the number of neural progenitor cells in the anterior DG [17]. Similarly, other pathological studies examining postmortem samples of individuals with MDD have reported that compared to untreated individuals, those treated with antidepressants have a larger number of granule neurons in the mid and anterior DG regions as well as larger volumes in the mid DG region [18]. Collectively, these studies suggest that neurogenesis in the DG may occur in the anterior-mid region (i.e. DG-head) rather than in the posterior region (i.e. DG-body). Hence, our finding of increased DG-head volume after BL exposure might reflect a pathological alteration in the DG. Additionally, our results showed a significant increase in the volume of the left DG-head but not in that of the right DG-head. These results could be due to the effect of the dominant hemisphere since all patients in this study were right-handed. Additionally, this left lateralization was consistent with our prior observation that 4 weeks of BL in healthy individuals increased the volume of the left hippocampal DG-head [7]. However, the possible influence of mood stabilizers such as lithium and antidepressants on the increased DG volume cannot be ruled out entirely. Nevertheless, the patients had maintained their psychotropic medication regimen during the study period as well as for at least 1 month before the study initiation. Additionally, we performed a subgroup analysis that excluded patients who were on lithium therapy and revealed a significant effect of time and group on the volumes of left DG-head and left DG-total. Thus, although the augmentative effect of BL on psychotropic medications cannot be entirely discounted, the impact of these medications on DG volume during the study period was likely minimal. Furthermore, we established a control group that was treated with dim light, and the group and time interactions between the BL and DL groups suggested that the increase in DG volume may have been caused by the intensity of the light rather than other factors. The enrollment of participants with mood disorders led to this study including two mixed diagnosis cases of MDD and BP, which could potentially represent different pathologies. However, their diagnoses were adjusted as covariates, and the significant effects of time and group were shown to persist in the left DG-head and left DG-total. Therefore, BLT may increase the volume of the left DG in patients with mood disorders, irrespective of the specific diagnosis of MDD or BP.

With regard to the impact of light on clinical symptoms, both the BL and DL groups showed efficacy in ameliorating depressive symptom. However, no significant differences were detected between time and group and the HAM-D and VAS of mood scores. This could be because the patients in this study exhibited mild depression, which could explain the modest decrease in scores and the absence of a significant difference. In a randomized controlled trial by Sit et al. that used light therapy for BP, the DL group demonstrated reduced HAM-D scores after 6 weeks, with 20% of the patients achieving remission [19]. Our findings suggest a similar effect on depressive symptoms in the DL control group, which could account for the lack of a significant difference in the group-time interaction. Furthermore, BLT exhibited effectiveness in decreasing YMRS scores. However, the mean baseline score of 1.4 points suggests that the participants were not in a hypomanic or manic state. Although the mean score after 4 weeks of intervention showed a significant decrease to 0.5 points, it was not clinically meaningful. In addition, no apparent side effects were observed in the BL group during the study period, indicating that BLT was a safe and well-tolerated intervention.

We posited that the increase in the volume of the left hippocampal DG (expressed as %Δ) would correspond with a reduction in the depression scores on the questionnaires. However, we did not observe a correlation with the HAM-D or BDI scores. This may be attributed to the fact that the mild depressive symptoms exhibited by the patients in this study did not generate a significant difference in scores and the %Δ detected in the HAM-D and BDI scores was relatively small. Nevertheless, a positive correlation was observed between the %Δ in the volume of the left DG-total and the %Δ in the VAS of mood scores in the BL group. This finding suggests that an increase in the volume of the left DG is linked to a significant improvement in mood. The DL group also showed a significant positive correlation between the %Δ in the volume of the left DG-body and the %Δ in the HAM-D scores. However, the DL group did not demonstrate an increase in the volume of the left-DG body after the intervention, which suggests that this significant correlation was coincidental.

Our current study has some limitations. First, the sample size was relatively small, which may limit the generalisability of our findings. Second, our results were based on volumetric changes in the DG, which are not a direct indicator of neurogenesis. This is because changes in the DG volume may arise not only from neurogenesis but also from enhancements in neuronal atrophy and synaptogenesis via dendritic reshaping. Third, although participants were instructed to maintain their lifestyle unchanged throughout the study’s duration at the inception of the study, it was difficult to completely exclude the effects of daily behaviors such as diet, exercise, and sleep on the hippocampal DG.

In conclusion, our study results suggest that BLT exhibits a dose-response effect, wherein light exposure of a higher intensity is more efficacious than that of a lower intensity in terms of increasing the volume of the left DG and alleviating depression symptoms. Thus, BLT may trigger neurogenesis in the left DG of patients with mood disorders, indicating a novel mechanism of action of BLT. Further studies with large sample sizes are needed to confirm these findings.

### Supplementary information

Supplementary Information

## Data Availability

The data supporting the conclusions of this research are available from the corresponding author, H. Hirakawa, upon reasonable request.
