# Supplementary material for: Increased volume of the left hippocampal dentate gyrus after 4 weeks of bright light exposure in patients with mood disorders: a randomized controlled study
Source: Transl Psychiatry. 2023 Dec 15;13:394. doi: 10.1038/s41398-023-02688-9 (PMC10724173; doi:10.1038/s41398-023-02688-9)
Supplement: Supplementary file 1 — Supplementary Information [file 41398_2023_2688_MOESM1_ESM.docx]

|  | Patients with mood disorder (n=24) |
| --- | --- |
| Age mean (s.d., range) | 38.0 (11.5, 41) |
| Sex (Male, Female) | 12, 12 |
| Diagnosis | MDD: 17, BP: 7 |
| Season | Spring:2, Summer:7, Autumn:7, Winter:8 |
| HAM-D (s.d., range) | 9.5 (3.8, 15) |
| BDI (s.d., range) | 19.8 (10.2, 36) |
| YMRS (s.d., range) | 1.1 (1.2, 4) |
| VAS of mood (s.d., range) | 44.0 (14.3, 58.0) |
|  |  |
| Baseline DG Volume mm^3^ mean (s.d., range) |  |
| Left DG-head | 154.8 (20.2, 90.2) |
| Left DG-body | 131.9 (17.7, 64.1) |
| Left DG-total | 286.8 (33.1, 133.2) |
| Right DG-head | 160.0 (18.2, 72.1) |
| Right DG-body | 135.8 (17.6, 69.0) |
| Right DG-total | 295.8 (30.5, 125.6) |

**Supplementary Table 1. The demographic characteristics of all patients with mood disorder**

Abbreviation; Beck Depression Inventory: BDI, Bipolar disorder: BP, Dentate gyrus: DG, Hamilton Depression Rating Scale: HAM-D, Major Depressive Disorder: MDD, Standard deviation: s.d., Visual Analog Scale: VAS, Young Mania Rating Scale: YMRS

*Indicates that the p values < 0.05, ** Indicates that the p values < 0.01

|  | Left DG-head | Left DG-body | Left DG-total | Right DG-head | Right DG-body | Right DG-total |
| --- | --- | --- | --- | --- | --- | --- |
| age | r = -0.06,  p = 0.79 | r = 0.1,  p = 0.64 | r = 0.07,  p = 0.73 | r = 0.31,  p = 0.13 | r = 0.28,  p = 0.18 | r = -0.3,  p = 0.16 |

**Supplementary Table 2. The correlation between age and the volume of the hippocampal dentate gyrus**

Abbreviation; Dentate gyrus: DG

*Indicates that the p values < 0.05, ** Indicates that the p values < 0.01

|  | Patients with mood disorder (n=24) | | p value |
| --- | --- | --- | --- |
|  | Male (n=12) | Female (n=12) |  |
| Age mean (s.d.) | 40.1 (12.1) | 35.9 (11.0) | 0.39 |
|  |  |  |  |
| Baseline DG Volume mm^3^ mean (s.d.) |  |  |  |
| Left DG-head | 155.2 (19.0) | 154.4 (22.2) | 0.93 |
| Left DG-body | 128.1 (20.9) | 135.8 (13.8) | 0.15 |
| Left DG-total | 283.3 (35.7) | 290.2 (31.5) | 0.62 |
| Right DG-head | 162.0 (14.4) | 158.1 (21.9) | 0.61 |
| Right DG-body | 135.2 (19.2) | 136.4 (16.7) | 0.86 |
| Right DG-total | 297.2 (25.1) | 294.5 (36.2) | 0.42 |

**Supplementary Table 3. The age and the volume of the hippocampal dentate gyrus of patients with mood disorder divided by the sex**

Abbreviation; Dentate gyrus: DG

*Indicates that the p values < 0.05, ** Indicates that the p values < 0.01

|  | Type III  sum of squares | dF | Mean square | F | Partial η2 | Effect size f | p value |
| --- | --- | --- | --- | --- | --- | --- | --- |
| **Q****uestionnaire** |  |  |  |  |  |  |  |
| **HAM-D** |  |  |  |  |  |  |  |
| Time | 123.5 | 1 | 123.5 | 23.8 | 0.52 | 1.04 | 0.0001 ** |
| Group | 35.0 | 1 | 35.0 | 1.6 | 0.07 | 0.27 | 0.23 |
| Time*Group | 11.0 | 1 | 11.0 | 2.1 | 0.09 | 0.31 | 0.16 |
| **BDI** |  |  |  |  |  |  |  |
| Time | 939.3 | 1 | 939.3 | 63.1 | 0.74 | 1.69 | 0.0000001 ** |
| Group | 243.0 | 1 | 243.0 | 1.4 | 0.06 | 0.25 | 0.24 |
| Time*Group | 56.3 | 1 | 56.3 | 3.8 | 0.15 | 0.42 | 0.06 |
| **YMRS** |  |  |  |  |  |  |  |
| Time | 4.7 | 1 | 4.7 | 9.7 | 0.3 | 0.65 | 0.01 ** |
| Group | 0.2 | 1 | 0.2 | 0.1 | 0.005 | 0.07 | 0.74 |
| Time*Group | 0.19 | 1 | 0.2 | 0.39 | 0.02 | 0.14 | 0.54 |
| **VAS** |  |  |  |  |  |  |  |
| Time | 1868.8 | 1 | 1868.8 | 22.0 | 0.5 | 1.0 | 0.0001 ** |
| Group | 671.3 | 1 | 671.3 | 2.4 | 0.1 | 0.33 | 0.14 |
| Time*Group | 245.3 | 1 | 245.3 | 2.9 | 0.12 | 0.37 | 0.1 |
|  |  |  |  |  |  |  |  |
| **Adjusted age, sex, and log-transformed environmental light as covariates** | | | | | | | |
| **HAM-D** |  |  |  |  |  |  |  |
| Time | 3.7 | 1 | 3.7 | 0.75 | 0.04 | 0.2 | 0.39 |
| Group | 21.7 | 1 | 21.7 | 0.89 | 0.05 | 0.23 | 0.39 |
| Time*Group | 6.73 | 1 | 6.73 | 1.38 | 0.07 | 0.27 | 0.26 |
| **BDI** |  |  |  |  |  |  |  |
| Time | 0.2 | 1 | 0.2 | 0.02 | 0.001 | 0.03 | 0.9 |
| Group | 62.3 | 1 | 62.3 | 0.37 | 0.02 | 0.14 | 0.55 |
| Time*Group | 43.4 | 1 | 43.4 | 2.65 | 0.12 | 0.37 | 0.12 |
| **YMRS** |  |  |  |  |  |  |  |
| Time | 0.5 | 1 | 0.5 | 1.1 | 0.05 | 0.23 | 0.32 |
| Group | 0.03 | 1 | 0.03 | 0.02 | 0.001 | 0.03 | 0.89 |
| Time*Group | 0.03 | 1 | 0.03 | 0.06 | 0.003 | 0.05 | 0.81 |
| **VAS** |  |  |  |  |  |  |  |
| Time | 334.1 | 1 | 334.1 | 4.6 | 0.2 | 0.5 | 0.04 * |
| Group | 528.5 | 1 | 528.5 | 1.7 | 0.08 | 0.29 | 0.21 |
| Time*Group | 78 | 1 | 78 | 1.1 | 0.05 | 0.23 | 0.31 |
|  |  |  |  |  |  |  |  |
| **Adjusted age, sex, log-transformed environmental light, diagnoses as covariates** | | | | | | | |
| **HAM-D** |  |  |  |  |  |  |  |
| Time | 1.2 | 1 | 1.2 | 0.2 | 0.01 | 0.1 | 0.63 |
| Group | 20.2 | 1 | 20.2 | 0.8 | 0.04 | 0.2 | 0.39 |
| Time*Group | 5.67 | 1 | 5.67 | 1.1 | 0.06 | 0.25 | 0.31 |
| **BDI** |  |  |  |  |  |  |  |
| Time | 0.006 | 1 | 0.006 | 0.0003 | 0.00002 | 0.004 | 0.99 |
| Group | 58.7 | 1 | 58.7 | 2.2 | 0.1 | 0.33 | 0.15 |
| Time*Group | 40.5 | 1 | 40.5 | 2.35 | 0.12 | 0.37 | 0.14 |
| **YMRS** |  |  |  |  |  |  |  |
| Time | 0.85 | 1 | 0.85 | 1.7 | 0.09 | 0.31 | 0.21 |
| Group | 0.001 | 1 | 0.001 | 0.001 | 0.00005 | 0.007 | 0.98 |
| Time*Group | 0.08 | 1 | 0.08 | 0.15 | 0.008 | 0.09 | 0.7 |
| **VAS** |  |  |  |  |  |  |  |
| Time | 293.6 | 1 | 293.6 | 3.9 | 0.18 | 0.47 | 0.07 |
| Group | 717.8 | 1 | 717.8 | 2.4 | 0.12 | 0.37 | 0.14 |
| Time*Group | 85.8 | 1 | 85.8 | 1.1 | 0.06 | 0.25 | 0.3 |
|  |  |  |  |  |  |  |  |
| **DG Volume** |  |  |  |  |  |  |  |
| **Left DG-head** |  |  |  |  |  |  |  |
| Time | 17.4 | 1 | 17.4 | 3.2 | 0.13 | 0.39 | 0.09 |
| Group | 1.4 | 1 | 1.4 | 0.002 | 0.0001 | 0.01 | 0.97 |
| Time*Group | 62.9 | 1 | 62.9 | 11.6 | 0.35 | 0.73 | 0.003 ** |
| **Left DG-body** |  |  |  |  |  |  |  |
| Time | 0.01 | 1 | 0.01 | 0.002 | 0.0001 | 0.01 | 0.97 |
| Group | 0.47 | 1 | 0.47 | 0.001 | 0.00004 | 0.006 | 0.98 |
| Time*Group | 1.92 | 1 | 1.92 | 0.3 | 0.01 | 0.1 | 0.59 |
| **Left DG-total** |  |  |  |  |  |  |  |
| Time | 16.5 | 1 | 16.5 | 1.2 | 0.05 | 0.23 | 0.28 |
| Group | 0.23 | 1 | 0.23 | 0.0001 | 0.000005 | 0.002 | 0.99 |
| Time*Group | 86.8 | 1 | 86.8 | 6.5 | 0.23 | 0.55 | 0.02 * |
| **Right DG-head** |  |  |  |  |  |  |  |
| Time | 11.0 | 1 | 11.0 | 1.2 | 0.05 | 0.23 | 0.29 |
| Group | 57.7 | 1 | 57.7 | 0.09 | 0.004 | 0.06 | 0.77 |
| Time*Group | 0.08 | 1 | 0.08 | 0.01 | 0.0004 | 0.02 | 0.93 |
| **Right DG-body** |  |  |  |  |  |  |  |
| Time | 10.4 | 1 | 10.4 | 0.6 | 0.03 | 0.18 | 0.44 |
| Group | 569.6 | 1 | 569.6 | 0.9 | 0.04 | 0.2 | 0.35 |
| Time*Group | 0.61 | 1 | 0.61 | 0.04 | 0.002 | 0.04 | 0.85 |
| **Right DG-total** |  |  |  |  |  |  |  |
| Time | 42.7 | 1 | 42.7 | 1.6 | 0.07 | 0.27 | 0.22 |
| Group | 989.7 | 1 | 989.7 | 0.5 | 0.02 | 0.14 | 0.47 |
| Time*Group | 0.24 | 1 | 0.24 | 0.01 | 0.0004 | 0.02 | 0.93 |
|  |  |  |  |  |  |  |  |
| **Adjusted age, sex, and log-transformed environmental light as covariates** | | | | | | | |
| **Left DG-head** |  |  |  |  |  |  |  |
| Time | 2.1 | 1 | 2.1 | 0.36 | 0.02 | 0.14 | 0.56 |
| Group | 41.2 | 1 | 41.2 | 0.04 | 0.002 | 0.04 | 0.84 |
| Time*Group | 66.1 | 1 | 66.1 | 11.3 | 0.37 | 0.77 | 0.003 ** |
| **Left DG-body** |  |  |  |  |  |  |  |
| Time | 1.05 | 1 | 1.05 | 0.14 | 0.007 | 0.08 | 0.71 |
| Group | 74.8 | 1 | 74.8 | 0.12 | 0.006 | 0.08 | 0.73 |
| Time*Group | 1.94 | 1 | 1.94 | 0.27 | 0.01 | 0.1 | 0.61 |
| **Left DG-total** |  |  |  |  |  |  |  |
| Time | 6.1 | 1 | 6.1 | 0.4 | 0.02 | 0.14 | 0.53 |
| Group | 5.0 | 1 | 5.0 | 0.002 | 0.0001 | 0.01 | 0.97 |
| Time*Group | 90.7 | 1 | 90.7 | 6 | 0.24 | 0.56 | 0.02 * |
| **Right DG-head** |  |  |  |  |  |  |  |
| Time | 31.5 | 1 | 31.5 | 3.7 | 0.16 | 0.44 | 0.07 |
| Group | 604.2 | 1 | 604.2 | 1.0 | 0.05 | 0.23 | 0.32 |
| Time*Group | 6.5 | 1 | 6.5 | 0.77 | 0.04 | 0.2 | 0.39 |
| **Right DG-body** |  |  |  |  |  |  |  |
| Time | 10.3 | 1 | 10.3 | 0.6 | 0.03 | 0.18 | 0.44 |
| Group | 882.7 | 1 | 882.7 | 1.5 | 0.07 | 0.27 | 0.24 |
| Time*Group | 0.002 | 1 | 0.002 | 0.0001 | 0.000007 | 0.003 | 0.99 |
| **Right DG-total** |  |  |  |  |  |  |  |
| Time | 77.8 | 1 | 77.8 | 3.2 | 0.15 | 0.42 | 0.09 |
| Group | 2947.5 | 1 | 2947.5 | 1.9 | 0.09 | 0.31 | 0.18 |
| Time*Group | 6.3 | 1 | 6.3 | 0.26 | 0.01 | 0.1 | 0.62 |
|  |  |  |  |  |  |  |  |
| **Adjusted age, sex, log-transformed environmental light, diagnoses as covariates** | | | | | | | |
| **Left DG-head** |  |  |  |  |  |  |  |
| Time | 0.17 | 1 | 0.17 | 0.03 | 0.002 | 0.04 | 0.87 |
| Group | 7.8 | 1 | 7.8 | 0.01 | 0.01 | 0.1 | 0.93 |
| Time*Group | 68.2 | 1 | 68.2 | 11.3 | 0.39 | 0.8 | 0.003 ** |
| **Left DG-body** |  |  |  |  |  |  |  |
| Time | 2.97 | 1 | 2.97 | 0.39 | 0.02 | 0.14 | 0.54 |
| Group | 137.2 | 1 | 137.2 | 0.22 | 0.01 | 0.1 | 0.65 |
| Time*Group | 1.17 | 1 | 1.17 | 0.16 | 0.01 | 0.1 | 0.7 |
| **Left DG-total** |  |  |  |  |  |  |  |
| Time | 4.6 | 1 | 4.6 | 0.29 | 0.02 | 0.14 | 0.6 |
| Group | 79.5 | 1 | 79.5 | 0.03 | 0.002 | 0.04 | 0.86 |
| Time*Group | 87.3 | 1 | 87.3 | 5.49 | 0.23 | 0.55 | 0.03 * |
| **Right DG-head** |  |  |  |  |  |  |  |
| Time | 27.4 | 1 | 27.4 | 3.1 | 0.15 | 0.42 | 0.1 |
| Group | 493.7 | 1 | 493.7 | 0.8 | 0.04 | 0.2 | 0.38 |
| Time*Group | 5.47 | 1 | 5.47 | 0.62 | 0.03 | 0.18 | 0.44 |
| **Right DG-body** |  |  |  |  |  |  |  |
| Time | 19.8 | 1 | 19.8 | 1.2 | 0.06 | 0.25 | 0.29 |
| Group | 628.6 | 1 | 628.6 | 1.0 | 0.06 | 0.25 | 0.32 |
| Time*Group | 0.4 | 1 | 0.4 | 0.24 | 0.01 | 0.1 | 0.88 |
| **Right DG-total** |  |  |  |  |  |  |  |
| Time | 93.8 | 1 | 93.8 | 3.8 | 0.18 | 0.47 | 0.07 |
| Group | 2236.5 | 1 | 2236.5 | 1.5 | 0.07 | 0.27 | 0.24 |
| Time*Group | 2.91 | 1 | 2.91 | 0.12 | 0.007 | 0.08 | 0.73 |

**Supplementary Table 4. The effects of different light exposures (i.e. bright light group and dim light group) and time effect on DG volumes and questionnaire in patients with mood disorder.**

Abbreviation; Beck Depression Inventory: BDI, Dentate gyrus: DG, Hamilton Depression Rating Scale: HAM-D, Visual Analog Scale: VAS, Young Mania Rating Scale: YMRS

*Indicates that the p values < 0.05, ** Indicates that the p values < 0.01

|  | Type III  sum of squares | dF | Mean square | F | Partial η2 | Effect size f | p value |
| --- | --- | --- | --- | --- | --- | --- | --- |
| **DG Volume** |  |  |  |  |  |  |  |
| **Left DG-head** |  |  |  |  |  |  |  |
| Time | 12.8 | 1 | 12.8 | 2.4 | 0.13 | 0.39 | 0.14 |
| Group | 9.9 | 1 | 9.9 | 0.01 | 0.001 | 0.03 | 0.91 |
| Time*Group | 72 | 1 | 72 | 13.7 | 0.45 | 0.9 | 0.002 ** |
| **Left DG-body** |  |  |  |  |  |  |  |
| Time | 2.2 | 1 | 2.2 | 0.3 | 0.02 | 0.14 | 0.59 |
| Group | 265.8 | 1 | 265.8 | 0.4 | 0.02 | 0.14 | 0.53 |
| Time*Group | 1.37 | 1 | 1.37 | 0.19 | 0.01 | 0.1 | 0.67 |
| **Left DG-total** |  |  |  |  |  |  |  |
| Time | 4.4 | 1 | 4.4 | 0.3 | 0.02 | 0.14 | 0.57 |
| Group | 173.0 | 1 | 173.0 | 0.08 | 0.005 | 0.07 | 0.78 |
| Time*Group | 93.3 | 1 | 93.3 | 7.16 | 0.3 | 0.65 | 0.02 * |
| **Right DG-head** |  |  |  |  |  |  |  |
| Time | 4.4 | 1 | 4.4 | 0.41 | 0.02 | 0.14 | 0.53 |
| Group | 14.8 | 1 | 14.8 | 0.02 | 0.001 | 0.03 | 0.88 |
| Time*Group | 0.69 | 1 | 0.69 | 0.07 | 0.004 | 0.06 | 0.8 |
| **Right DG-body** |  |  |  |  |  |  |  |
| Time | 7.9 | 1 | 7.9 | 0.43 | 0.02 | 0.14 | 0.52 |
| Group | 34.9 | 1 | 34.9 | 0.06 | 0.003 | 0.05 | 0.82 |
| Time*Group | 0.12 | 1 | 0.12 | 0.006 | 0.0003 | 0.02 | 0.94 |
| **Right DG-total** |  |  |  |  |  |  |  |
| Time | 24.0 | 1 | 24.0 | 0.8 | 0.05 | 0.23 | 0.38 |
| Group | 95.1 | 1 | 95.1 | 0.06 | 0.003 | 0.05 | 0.81 |
| Time*Group | 0.24 | 1 | 0.24 | 0.008 | 0.0004 | 0.02 | 0.93 |
|  |  |  |  |  |  |  |  |
| **Adjusted age, sex, and log-transformed environmental light as covariates** | | | | | | | |
| **Left DG-head** |  |  |  |  |  |  |  |
| Time | 1.4 | 1 | 1.4 | 0.24 | 0.02 | 0.14 | 0.63 |
| Group | 4.1 | 1 | 4.1 | 0.04 | 0.0003 | 0.05 | 0.95 |
| Time*Group | 79.8 | 1 | 79.8 | 13.9 | 0.5 | 1 | 0.002 ** |
| **Left DG-body** |  |  |  |  |  |  |  |
| Time | 4.1 | 1 | 4.1 | 0.5 | 0.04 | 0.2 | 0.49 |
| Group | 531.1 | 1 | 531.1 | 0.8 | 0.06 | 0.25 | 0.38 |
| Time*Group | 0.71 | 1 | 0.71 | 0.09 | 0.006 | 0.08 | 0.77 |
| **Left DG-total** |  |  |  |  |  |  |  |
| Time | 10.2 | 1 | 10.2 | 0.68 | 0.05 | 0.23 | 0.42 |
| Group | 441.8 | 1 | 441.8 | 0.18 | 0.01 | 0.1 | 0.68 |
| Time*Group | 95.6 | 1 | 95.6 | 6.35 | 0.31 | 0.67 | 0.02 * |
| **Right DG-head** |  |  |  |  |  |  |  |
| Time | 14.5 | 1 | 14.5 | 1.36 | 0.09 | 0.31 | 0.26 |
| Group | 81.0 | 1 | 81.0 | 0.13 | 0.009 | 0.1 | 0.73 |
| Time*Group | 7.71 | 1 | 7.71 | 0.72 | 0.05 | 0.23 | 0.41 |
| **Right DG-body** |  |  |  |  |  |  |  |
| Time | 4.4 | 1 | 4.4 | 0.22 | 0.02 | 0.14 | 0.65 |
| Group | 48.7 | 1 | 48.7 | 0.07 | 0.005 | 0.07 | 0.79 |
| Time*Group | 0.25 | 1 | 0.25 | 0.01 | 0.001 | 0.03 | 0.91 |
| **Right DG-total** |  |  |  |  |  |  |  |
| Time | 35.1 | 1 | 35.1 | 1.2 | 0.08 | 0.29 | 0.3 |
| Group | 255.3 | 1 | 255.3 | 0.17 | 0.01 | 0.1 | 0.69 |
| Time*Group | 10.7 | 1 | 10.7 | 0.36 | 0.03 | 0.18 | 0.56 |
|  |  |  |  |  |  |  |  |
| **Adjusted age, sex, log-transformed environmental light, diagnoses as covariates** | | | | | | | |
| **Left DG-head** |  |  |  |  |  |  |  |
| Time | 0.2 | 1 | 0.2 | 0.03 | 0.002 | 0.04 | 0.87 |
| Group | 3.5 | 1 | 3.5 | 0.005 | 0.0004 | 0.02 | 0.94 |
| Time*Group | 81.2 | 1 | 81.2 | 13.5 | 0.51 | 1.02 | 0.003 ** |
| **Left DG-body** |  |  |  |  |  |  |  |
| Time | 1.8 | 1 | 1.8 | 0.2 | 0.02 | 0.14 | 0.66 |
| Group | 655.2 | 1 | 655.2 | 1.2 | 0.08 | 0.29 | 0.3 |
| Time*Group | 0.83 | 1 | 0.83 | 0.1 | 0.007 | 0.08 | 0.76 |
| **Left DG-total** |  |  |  |  |  |  |  |
| Time | 3.0 | 1 | 3.0 | 0.2 | 0.02 | 0.14 | 0.67 |
| Group | 753.9 | 1 | 753.9 | 0.43 | 0.03 | 0.18 | 0.52 |
| Time*Group | 98.4 | 1 | 98.4 | 6.29 | 0.33 | 0.7 | 0.03 * |
| **Right DG-head** |  |  |  |  |  |  |  |
| Time | 24.1 | 1 | 24.1 | 2.3 | 0.15 | 0.42 | 0.16 |
| Group | 56.1 | 1 | 56.1 | 0.09 | 0.007 | 0.08 | 0.77 |
| Time*Group | 6.6 | 1 | 6.6 | 0.62 | 0.05 | 0.23 | 0.45 |
| **Right DG-body** |  |  |  |  |  |  |  |
| Time | 12.5 | 1 | 12.5 | 0.61 | 0.04 | 0.2 | 0.45 |
| Group | 26.5 | 1 | 26.5 | 0.04 | 0.003 | 0.05 | 0.84 |
| Time*Group | 0.08 | 1 | 0.08 | 0.004 | 0.0002 | 0.01 | 0.95 |
| **Right DG-total** |  |  |  |  |  |  |  |
| Time | 71.4 | 1 | 71.4 | 2.6 | 0.17 | 0.45 | 0.13 |
| Group | 159.6 | 1 | 159.6 | 0.1 | 0.009 | 0.1 | 0.74 |
| Time*Group | 8.1 | 1 | 8.1 | 0.29 | 0.02 | 0.14 | 0.6 |

**Supplementary Table 5. The effects of different light exposures (i.e. bright light group and dim light group) and time effect on DG volumes in patients with mood disorder excluding those receiving lithium.**

Abbreviation; Beck Depression Inventory: BDI, Dentate gyrus: DG, Hamilton Depression Rating Scale: HAM-D, Visual Analog Scale: VAS, Young Mania Rating Scale: YMRS

*Indicates that the p values < 0.05, ** Indicates that the p values < 0.01

|  | Patients with mood disorder excluding those receiving lithium | | | | | | | | |  |
| --- | --- | --- | --- | --- | --- | --- | --- | --- | --- | --- |
|  | BL group pre | BL group post | 95 % CI | d | p value | DL group pre | DL group post | 95 % CI | d | p value |
| **DG Volume** **mm^3^** mean (s.d.) |  |  |  |  |  |  |  |  |  |  |
| Left DG-head | 155.6 (25.6) | 159.6 (25.0) | -6.0 - -1.9 | 1.5 | 0.002 ** | 157.4 (13.0) | 155.8 (13.4) | -1.1 - 4.2 | 0.43 | 0.21 |
| Left DG-body | 130.2 (16.4) | 130.1 (16.6) | -1.0 - 1.2 | 0.07 | 0.84 | 135.9 (20.49 | 135.0 (17.9) | -2.7 - 4.4 | 0.17 | 0.6 |
| Left DG-total | 285.8 (37.3) | 289.6 (37.2) | -6.0 - -1.6 | 1.32 | 0.004 ** | 293.2 (30.1) | 290.8 (28.3) | -2.2 - 7.1 | 0.38 | 0.26 |
| Right DG-head | 160.7 (22.4) | 161.1 (22.4) | -2.8 - 2.0 | 0.13 | 0.71 | 159.1 (12.6) | 160.1 (14.4) | -5.0 -3.1 | 0.17 | 0.61 |
| Right DG-body | 136.4 (16.6) | 137.4 (15.1) | -5.5 - 3.5 | 0.18 | 0.61 | 134.6 (18.6) | 135.4 (20.1) | -5.3 - 3.7 | 0.13 | 0.7 |
| Right DG-total | 297.1 (34.1) | 298.5 (30.7) | -6.8 - 3.9 | 0.21 | 0.55 | 293.7 (24.1) | 295.5 (26.6) | -7.8 - 4.3 | 0.21 | 0.53 |

**Supplementary Table 6. Volumetric change of hippocampal dental gyrus after bright or dim light exposure in patients with mood disorder excluding those receiving lithium.**

Abbreviation; Bright light exposure group: BL group, Dentate gyrus: DG, Dim light exposure group: DL group, Standard deviation: s.d.,

*Indicates that the p values < 0.05, ** Indicates that the p values < 0.01

|  | Type III  sum of squares | dF | Mean square | F | Partial η2 | Effect size f | p value |
| --- | --- | --- | --- | --- | --- | --- | --- |
| **Subscale of HAM-D** |  |  |  |  |  |  |  |
| **Depressed mood** |  |  |  |  |  |  |  |
| Time | 3.5 | 1 | 3.5 | 8.8 | 0.29 | 0.64 | 0.01 * |
| Group | 1.7 | 1 | 1.7 | 4 | 0.15 | 0.42 | 0.06 |
| Time*Group | 0.2 | 1 | 0.2 | 0.5 | 0.02 | 0.14 | 0.5 |
| **Work and interest** |  |  |  |  |  |  |  |
| Time | 1.3 | 1 | 1.3 | 6.8 | 0.24 | 0.56 | 0.02 |
| Group | 0.1 | 1 | 0.1 | 0.2 | 0.01 | 0.1 | 0.65 |
| Time*Group | 1.3 | 1 | 1.3 | 6.8 | 0.24 | 0.56 | 0.02 * |
|  |  |  |  |  |  |  |  |
| **Adjusted age, sex, and log-transformed environmental light as covariates** | | | | | | | |
| **Subscale of HAM-D** |  |  |  |  |  |  |  |
| **Depressed mood** |  |  |  |  |  |  |  |
| Time | 0.06 | 1 | 0.06 | 0.2 | 0.01 | 0.1 | 0.69 |
| Group | 1.5 | 1 | 1.5 | 3.6 | 0.16 | 0.44 | 0.07 |
| Time*Group | 0.17 | 1 | 0.17 | 0.4 | 0.02 | 0.14 | 0.51 |
| **Work and interest** |  |  |  |  |  |  |  |
| Time | 0.1 | 1 | 0.1 | 0.5 | 0.03 | 0.18 | 0.47 |
| Group | 0.04 | 1 | 0.04 | 0.1 | 0.01 | 0.1 | 0.74 |
| Time*Group | 1.6 | 1 | 1.6 | 9.6 | 0.34 | 0.71 | 0.01 * |
|  |  |  |  |  |  |  |  |
| **Adjusted age, sex, log-transformed environmental light, diagnoses as covariates** | | | | | | | |
| **Subscale of HAM-D** |  |  |  |  |  |  |  |
| **Depressed mood** |  |  |  |  |  |  |  |
| Time | 0.1 | 1 | 0.1 | 0.2 | 0.01 | 0.1 | 0.63 |
| Group | 1.2 | 1 | 1.2 | 2.9 | 0.14 | 0.4 | 0.11 |
| Time*Group | 0.2 | 1 | 0.2 | 0.5 | 0.03 | 0.18 | 0.5 |
| **Work and interest** |  |  |  |  |  |  |  |
| Time | 0.16 | 1 | 0.16 | 0.9 | 0.05 | 0.23 | 0.35 |
| Group | 0.05 | 1 | 0.05 | 0.1 | 0.01 | 0.1 | 0.71 |
| Time*Group | 1.5 | 1 | 1.5 | 8.3 | 0.32 | 0.69 | 0.01 * |

**Supplementary Table 7. The effects of different light exposures (i.e. bright light group and dim light group) and time effect on the subscale of Hamilton Depression Rating Scale in patients with mood disorder.**

Abbreviation; Hamilton Depression Rating Scale: HAM-D

*Indicates that the p values < 0.05, ** Indicates that the p values < 0.01

|  | Patients with the mood disorder | | | | | | | | |  |
| --- | --- | --- | --- | --- | --- | --- | --- | --- | --- | --- |
|  | BL group pre | BL group post | 95 % CI | Cohen d | p value | DL group pre | DL group post | 95 % CI | Cohen d | p value |
| **Subscale of  HAM-D** |  |  |  |  |  |  |  |  |  |  |
| Depressed  mood | 0.8 (0.8) | 0.1 (0.3) | 0.2 - 1.2 | 1.17 | 0.01* | 1.0 (0.7) | 0.6 (0.7) | -0.2 -1.1 | 0.42 | 0.18 |
| Work and  interest | 1.3 (0.5) | 0.7 (0.7) | 0.1 - 1.2 | 1.19 | 0.03* | 1.1 (0.5) | 1.1 (0.5) | - | - | 1 |

**Supplementary Table 8. Subscale of Hamilton Depression Rating Scale change after bright or dim light exposure in patients with mood disorder**

Abbreviation; Bright light exposure group: BL group, Confidence interval: CI, Dim light exposure group: DL group, Hamilton Depression Rating Scale: HAM-D

*Indicates that the p values < 0.05, ** Indicates that the p values < 0.01
